# Prevalence of positive tuberculin skin test in a Brazilian sample of rheumatoid arthritis and spondylarthritis patients

**DOI:** 10.1590/1806-9282.20230725

**Published:** 2024-01-22

**Authors:** Beatriz Silva Lemes, Carina Albuquerque Roberto, André Rochinski Busanello, Bárbara Stadler Kahlow, Thelma Skare, Renato Nisihara

**Affiliations:** 1 Mackenzie Evangelical School of Medicine of Paraná – Curitiba (PR), Brazil.; 2 Mackenzie Evangelical University Hospital, Rheumatology Unit – Curitiba (PR), Brazil.

**Keywords:** Tuberculosis, Arthritis, rheumatoid, Spondylarthritis, Tuberculin test

## Abstract

**OBJECTIVE:**

Patients with rheumatic diseases have an increased risk of infections, especially tuberculosis. In this study, we aimed to recognize the positivity rate of tuberculosis skin test in patients with rheumatoid arthritis and spondyloarthritis and the characteristics of the patients with positive results.

**METHODS:**

Retrospective study of tuberculosis skin test results in patients followed from 2004 to 2021 in a single rheumatology unit. Data related to clinical and epidemiological features, along with treatment information referring to the period in which the tuberculosis skin test was performed, were collected from patients’ charts.

**RESULTS:**

A total of 723 tests were identified (448 tests in 269 rheumatoid arthritis patients and 275 in 174 spondyloarthritis patients). In the rheumatoid arthritis sample, 31/275 (11.5%) individuals had positive tests, and in the spondyloarthritis, 38/174 (21.8%) had positive tests. In the rheumatoid arthritis sample, patients with positive tuberculosis skin tests used a higher dose of methotrexate than those with negative results (median of 25 mg/week versus median of 20 mg/week respectively; p=0.02). In the spondyloarthritis sample, tuberculosis skin test positivity was associated with alcohol ingestion (13.1% versus 2.9% in users and non-users respectively; p=0.02) and sulfasalazine use (15.7% of positivity in users versus 5% in non-users; p=0.01).

**CONCLUSION:**

The tuberculosis skin test-positive prevalence in rheumatoid arthritis was lower than in the spondyloarthritis sample. Patients with rheumatoid arthritis using a higher dosage of methotrexate or with spondyloarthritis using sulfasalazine had more frequency of tuberculosis skin test positivity and should be carefully followed by the attending physician in order to avoid the appearance of full-blown tuberculosis.

## INTRODUCTION

Tuberculosis (TB) is a major origin of morbidity and mortality worldwide^1^. This infection is particularly prevalent among individuals using immunosuppressive medications and/or with immune dysregulation such as patients with rheumatic diseases and is of special concern among those using biological drugs^1^. Screening for latent tuberculosis infection (LTBI) is an effective way to recognize individuals who are at high risk of developing the active disease form and who may benefit from prophylactic treatment^2^. The use of isoniazid offers a 90% reduction in tuberculosis in those who are HIV-negative and complete the treatment^2^.

The LTBI screening has been historically done using the tuberculin skin test (TST), as it is a cheap test that does not require a laboratory, although it requires a trained operator to perform it^3^. Interferon-gamma release assays (IGRAs) have been used recently aiming to improve the diagnosis precision^2^. However, it is debated which one, TST or IGRAs, should be used to best predict latent infection and which one is more cost-effective^2^. The TST results show variabilities in the population according to the geographical background, as it may suffer the influence of earlier use of bacillus Calmette-Guérin (BCG) vaccination, and of infections from other environmental mycobacteria^4^.

In Brazil, only TSTs are available through the National Health System and hence, they are the most used in public rheumatology clinics^5^. In rheumatology, TST is routine when patients are about to start biological drugs, but no recommendations referring to the mandatory realization before the use of other immunosuppressants or about its reapplication are available nowadays. In this context, they are prescribed according to the physician’s judgment. When a positive test is detected, patients are directed to the basic units of the Health Public System for registration, tracking of contacts, and treatment. Brazil is a country with a high rate of tuberculosis infections: about 86.166 new cases of active tuberculosis infection have been notified in the country during 2020 without preference among sex or age group^6^. Due to this high prevalence, BCG vaccination is recommended in all newborns and it should be done as early as possible, preferably in the maternity, right after birth^7^. Nevertheless, the effect of BCG vaccination declines in individuals older than 30 years^8^.

In recent years, tracking latent tuberculosis in Brazil has been impaired due to the lack of purified protein derivative (PPD) to be used in the Mantoux test. In this situation, the assessment of latent infection was primarily based on clinical history, history of exposure to the infection, and image testing.

Herein, a retrospective study was done to recognize the positivity rate of TST in patients with rheumatoid arthritis (RA) and spondyloarthritis (SpA) and the demographic and/or treatment characteristics associated with positive results, aiming to provide data on at-risk patients who should be monitored more carefully.

## METHODS

### Ethical issues

This is a retrospective study approved by the local Committee of Ethics in Research (CAAE 38173120.8.0000.0103) under protocol number 4377896. Due to the retrospective nature of the study, the Committee of Ethics in Research waived the free and informed consent. All researchers signed a confidentiality agreement.

### Participants

A convenience sample was studied. All the charts from RA patients and SpA patients from a single rheumatology unit from Southern Brazil from 2004 to 2021 were reviewed for the results of TST. This is a rheumatology unit that cares for patients from the Public Health System. Data on TST results, medications used at the time of TST realization, and epidemiological data were extracted from the charts.

Inclusion criteria were as follows: patients of both sexes, over 18 years old with a confirmed diagnosis of RA or SpA, and with at least one TST result before the beginning of treatment for the rheumatic disease.

Exclusion criteria were as follows: patients who had lost follow-up during the study period or those with incomplete charts.

### Tuberculosis skin test

The TST was done by the Mantoux method. The reaction to intradermal tuberculin is of the delayed hypersensitivity type, which represents the reaction to a hapten, and is mediated by T cells and only produces reactions in the dermis when there is an infection with *Mycobacterium tuberculosis* (protocol Health Ministry from Brazil, 2014). Hypersensitivity was demonstrated by the presence of skin induration at the site of tuberculin injection (0.1 mL) after 48–72 h^5^.

In Brazil, the tuberculin used is PPD RT23 (Statens Serum Institut, Copenhagen/Denmark) at a dose of 0.1 mL, which contains two tuberculin units^9^. When induration was equal to or greater than 5 mm, the result was considered positive. The TST was carried out in the basic health unit where the patient was a resident.

### Statistical analyses

Data were studied through frequency and contingency tables. Data distribution was analyzed by the Shapiro-Wilk test. Nominal variables were expressed in percentage and the central tendency of numerical data was expressed in mean and standard deviation if the data were parametric and median or interquartile range if non-parametric. The Fisher’s and chi-squared tests were used to compare nominal data (gender, use of medications, tobacco and alcohol exposure, etc., in patients with positive and negative TST), and the Mann-Whitney and unpaired t tests were used for comparison of data with non-parametric and parametric distribution, respectively (to compare age, disease duration, and medication dose in TST-positive and TST-negative individuals). The adopted significance was 5%. Tests were performed using the GraphPad Prism version 8.0.0 software for Windows (GraphPad Software, San Diego, CA, USA,www.graphpad.com).

## RESULTS

**Figure 1.**
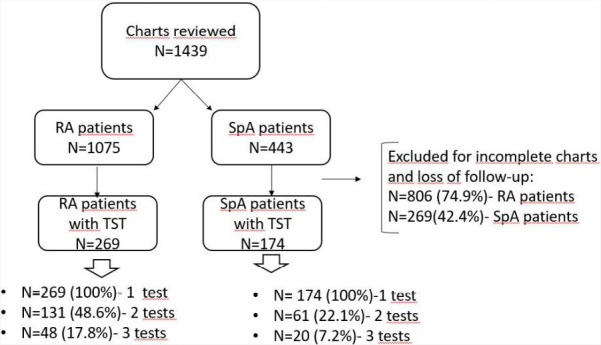
Diagram of chart’s selection for the study. N: number; RA: rheumatoid arthritis; SpA: spondylarthritis; TST: tuberculin skin test.

The studied sample was selected according to the flowchart shown in Figure 1.

Table 1 shows the main characteristics of the studied sample. In the 433 studied individuals, TST was positive in 69 (15.5%).

**Table 1.** Main characteristics of the studied sample (n=443 patients).

Female (n)	313/443 (70.6%)
Mean (SD) age at first TST (years)	55.2 (12.2)
Smokers (n)	130/443(29.3%)
Alcohol use (n)	15/ 443 (3.3%)
Median (IQR) disease duration at first TST (years)	11.0 (7.0–17.0)
Diabetes mellitus (n)	84/443 (18.9%)
Arterial hypertension (n)	203/443 (45.8%)

N: number; TST: tuberculin skin test; SD: standard deviation; IQR: interquartile range.

### Study of patients with rheumatoid arthritis

The main characteristics of the RA sample at first TST are described in Table 2.

**Table 2.** Main features of rheumatoid arthritis studied sample (n=269).

Mean (SD) age at first TST (years)	58.1 (10.7)
Smokers (n)	84 (31.2%)
Alcohol use	6 (2.2%)
Median (IQR) disease duration at first TST (years)	14 (9–19)
Rheumatoid factor (n)	159/263 (60.4%)
Diabetes mellitus (n)	52/269 (19.3%)
Arterial hypertension (n)	128/269 (47.5%)

N: number; TST: tuberculin skin test; IQR: interquartile range; SD: standard deviation.

A total of 448 TSTs were done in the 269 RA patients, and 31/269 (11.5%) individuals tested positive (21/31 or 67.7% in the first test, 7/31 or 22.6% in the second test, and 3/31 or 9.7% in the third test). The median interval between the first and second TSTs was 3 years (IQR=2–5), and between the second and third tests, it was 2.5 years (IQR=2–5). Once a patient was positive in one test, he was excluded from further analysis.

The comparison of TST-positive and TST-negative patients did not show differences in sex, tobacco or alcohol use, the presence of diabetes or hypertension, or rheumatoid factor positivity (all with p>0.05).

Table 3 displays the use of medication at the time of TST in positive and negative individuals showing that positive patients used higher dose of methotrexate than negative patients.

**Table 3.** Comparison of medications used at tuberculin skin test time in rheumatoid arthritis patients.

	Total n=448	TST-positive n=31	TST-negative n=417	p (^*^)
Prednisone (n)	265/448 (59.1%)	17/31 (54.8%)	248/417 (59.4%)	0.60
Median (IQR) prednisone dose/day	10 (5–10)	10 (5–10)	10 (5–10)	0.61
Sulfasalazine (n)	5/448 (1.1%)	1/31 (3.2%)	4/417 (0.9%)	0.30
Antimalarials (n)	69/448 (15.4%)	7/31 (22.5%)	62/417 (14.8%)	0.25
Methotrexate (n)	208/448 ( 46.4%)	17/31 (54.8%)	191/417 (45.8%)	0.33
Median (IQR) methotrexate dose (mg/week)	20 (15–25)	25 (22.5–25)	20 (15–25)	**0.02**
Leflunomide (n)	36/448 (8.0%)	4/31 (12.9%)	32/417 (7.6%)	0.29
Biological drugs (n)	53/448 (11.8%)	4/31 (12.9%)	49/417 (11.7%)	0.77

(*) refers to the comparison of TST-positive versus TST-negative. N: number; IQR: interquartile range; TST: tuberculin skin test. The statistically significant value is indicated in bold.

### Patients with spondylarthritis

Table 4 displays the main characteristics of SpA patients at first TST.

**Table 4.** Main features of spondylarthritis sample (n=174).

Male (n)	96 (55.1%)
Mean (SD) age at first TST (years)	50.7 (13.1)
Smokers (n)	46 (26.4%)
Alcohol use (n)	9 (5.1%)
Median (IQR) disease duration at first TST (years)	8 (5–14)
SpA subtype (n)	
Ankylosing spondylitis	134 (77.0%)
Psoriatic arthritis	23 (13.2%)
Others	17 (9.7%)
Diabetes mellitus (n)	32 (18.3%)
Arterial hypertension (n)	75 (43.1%)

N: number; TST: tuberculin skin test; IQR: interquartile range; SD: standard deviation; SpA: spondyloarthritis.

A total of 255 TSTs were done in 174 SpA patients, and 38/174 (21.8%) individuals tested positive. About 26/38 (68.4%) were positive in the first test, 7/38 (18.4%) in the second test, and 5/38 (13.1%) in the third test.

The median interval between the first and second tests was 5 years (IQR=2.7–8.2), and between the second and third tests, it was 9 years (IQR=8.5–14.0). Once a patient was positive in one test, he was excluded from further analysis.

TST-positive and TST-negative SpA patients did not differ in sex, tobacco use, and the presence of diabetes or hypertension (all with p>0.05). However, those using alcohol were more common in the TST-positive group than in the TST-negative group (13.1% versus 2.9%; p=0.02).

Table 5 shows the comparison of medications used by those with positive and negative TSTs when the test was done. This table shows that sulfasalazine was more commonly used in the TST-positive group which also had a trend toward higher methotrexate dose when compared with the negative TST individuals.

**Table 5.** Comparison of medications used at tuberculin skin test time in patients with spondylarthritis.

	Total n=255	TST-positive n=38	TST-negative n=217	p (^*^)
Prednisone (n)	13/255 (5.0%)	2 (5.2%)	11 (5.0%)	0.99
Sulfasalazine (n)	17/255 (6.6%)	6 (15.7%)	11 (5.0%)	0.01 (^**^)
Methotrexate (n)	45/255 (17.6%)	5 (13.1%)	40 (18.4%)	0.43
Median (IQR) methotrexate dose (mg/week)	20.0 (15.0–21.2)	25.0 (17.5–25.0)	20.0 (15.0–20.0)	0.08
Biological drugs (n)	44/255 (17.2%)	7 (18.4%)	37 (17.0%)	0.83

N: number; IQR: interquartile range; TST: tuberculin skin test.

(*) refers to the comparison of TST-positive versus TST-negative.

(**) OR=3.5; 95%CI 1.2–10.1. The statistically significant value is indicated in bold.

## DISCUSSION

This study shows that patients with SpA had higher positivity of TST than RA patients (21.8% versus 11.5%). In RA patients, the positivity of TST showed an association with methotrexate dosage, and in SpA, TST was more frequently found positive in those using alcohol and sulfasalazine.

The currently found TST positivity in RA patients is similar to those of two other studies from South America. Tamborenea et al.^10^ studied a sample of 105 RA patients from Uruguay and found a TST positivity in 12.4%, while Bautista-Molano et al.^11^ analyzed 261 patients from Colombia and detected positivity in 16%. Interestingly, in the first-mentioned study, the higher dosage of methotrexate was associated with tuberculin anergy while in the present findings, the higher methotrexate dose was associated with positive results. In the second-mentioned study, by Bautista-Molano et al.^11^, 100% of TST-positive patients were receiving methotrexate.

Patients with RA may have decreased responsiveness to TST by the disease itself, as their regulatory T cells, which have a role in the magnitude of the test, are decreased in number and function^12^. Therefore, it is reasonable to hypothesize that the immunosuppressive effect of MTX on lymphocytes would further increase the number of negative screening test. Methotrexate prevents folic acid activation through the inhibition of dihydrofolate reductase that limits DNA formation and cellular division, and has cytotoxic effects, causing cell death by apoptosis^13,14^. However, an interesting study in psoriasis patients^4^ showed an increase in positive tuberculin tests after the introduction of methotrexate treatment. In the same study, an increase in interferon (IFN)γ after the methotrexate introduction was also observed. TNFα and IFN γ are crucial for tuberculosis granuloma formation^15,16^. Aries Guillén et al.^17^ reported that, in rheumatic patients, methotrexate was associated with a twofold increase in TST positivity without associations with IGRA test results, postulating that methotrexate induces a false positivity. One explanation for this apparent paradox is that the cytotoxic effect of methotrexate, leading to mononuclear cell death, releases IFN-γ and TNF-α which are responsible for macrophage activation^4^.

Presently, SpA patients had a higher rate of TST positivity than RA patients. The risk of tuberculosis infection in the TNF blocker-naive SpAs group of individuals has been estimated to be 4.3-fold higher than that in the general population^18,19^. A considerable reduction in the tuberculosis frequency in patients receiving anti-TNF therapy has been observed recently as the screening and treatment for latent infection were applied as routine^18,20^. In addition, according to the Centers for Disease Control and Prevention (CDC) of the United States, the usual cutoff point of a positive test was reduced from 10 to 5 mm targeting a better control of the infection in this context^21,22^.

Another finding of this study was that SpA patients using sulfasalazine had more positive tests than those without it. According to Cantini et al.^23^, conventional immunomodulators used in rheumatology, except sulfasalazine, are associated with an increased risk of tuberculosis. Sulfasalazine is considered a drug with low immunosuppressive effect^24^, but it is one of the medications currently used in rheumatology that is associated with high chances of poor outcome in infection for SAR-COV-2^25^. So, it is possible to justify a higher prevalence of positive TSTs not only by an increased chance of tuberculosis infection but also by a good immune response to TST with drug use.

This study has several limitations: one of them is its retrospective nature and the other is that IGRA tests were not available in this population. The number of included patients was small, and taking into account the number of reviewed charts less than half had results of TST. Also, comorbidities were not considered and this may have influenced the obtained results. More studies with larger samples, prospective design, and IGRA tests are needed to better understand the repercussions of tuberculosis in rheumatic patients. However, this study does represent the real-life scenario in the Public Health Service in Brazil.

## CONCLUSION

The prevalence of positive TSTs in the RA sample was 11.5%, and in the SpA sample, it was 21.8%. RA patients using higher dose of methotrexate as well as SpA patients using alcohol and sulfasalazine had more positivity of TSTs. Individuals with these characteristics should be carefully followed in order to avoid the complete development of the disease.

## ETHICS APPROVAL

All procedures performed in studies involving human participants were in accordance with the ethical standards of the institutional research committee and with the 1964 Helsinki Declaration and its later amendments or comparable ethical standards. This study was approved by the Committee of Ethics in Research from Evangelic Mackenzie School of Medicine under protocol number 4.023.752.
